# The Impact of Short-Term, Intensive Antifolate Treatment (with Pyrimethamine and Sulfadoxine) and Antibiotics Followed by Long-Term, Secondary Antifolate Prophylaxis on the Rate of Toxoplasmic Retinochoroiditis Recurrence

**DOI:** 10.1371/journal.pntd.0004892

**Published:** 2016-08-19

**Authors:** Piotr K. Borkowski, Joanna Brydak-Godowska, Wojciech Basiak, Karolina Świtaj, Hanna Żarnowska-Prymek, Maria Olszyńska-Krowicka, Piotr Kajfasz, Daniel Rabczenko

**Affiliations:** 1 Former Department of Zoonoses and Tropical Diseases, Medical University of Warsaw, Poland, present Department of Infectious, Tropical Diseases and Hepatology, Medical University of Warsaw, Poland; 2 Department of Ophthalmology, Medical University of Warsaw, Poland; 3 Department-Centre for Monitoring and Analyses of Population Health Status, National Institute of Public Health—National Institute of Hygiene, Warsaw, Poland; Ohio State University, UNITED STATES

## Abstract

**Purpose:**

To assess the impact of intensive antifolate treatment, followed by secondary antifolate prophylaxis (A-SP) on the recurrence rate of toxoplasmic retinochoroiditis (TRC). To investigate whether there are any other factors potentially predisposing for recurrence.

**Material and Methods:**

A total of 637 medical records of TRC patients, who had been treated in the years 1994–2013 were reviewed. All patients were treated with pyrimethamine /sulfadoxine one 25mg/500mg tablet daily (P/S 25/500mg) for 21 days with a double loading dose for the first two days. From Day 2 the patients also received prednisone at a starting dose of 40mg and spiramycine 3 million IU three times daily, given for 10 days followed by azithromycin 500mg once daily for another 6 days. The analysis of the recurrence rate involved 352 patients who had completed 6-month secondary prophylaxis (P/S one 25 mg/500mg tablet twice a week).

**Results:**

When secondary antifolate prophylaxis (A-SP) was instituted immediately after the treatment for TRC, the probability of 3-year recurrence–free survival after the first course of A-SP was 90.9%. A recurrence was most likely approximately 3.5 years after the first treatment. A univariate Cox regression model demonstrated that a risk for recurrence was 2.82 times higher (p = 0.02) in patients with retinal scars. In the multivariate analysis, the risk for recurrence was 2.41 higher (p = 0.06). In patients with haemorrhagic lesions the risk for recurrence was lower, aRR = 0.17 (approaching borderline statistical significance p = 0.08).

**Conclusions:**

With the institution of A-SP of immediately after the intensive treatment for TRC, i.e. when a reactivation was most likely, there was no recurrence during A-SP. Following A-SP the recurrence rates were low and recurrence-free periods tended to be longer. The treatment regimen employed had a beneficial effect on the recurrence interval as it reduced and delayed the highest probability of recurrence.

## Author Summary

Ocular toxoplasmosis is the most common infectious disease of the posterior segment of the eye that could result in the loss of vision. Although modern knowledge of the mechanisms and interrelations which occur in the disease has recently significantly increased, there is the deficiency of randomized studies on large patient groups. Our retrospective work also does not have a control group. However, due to the highest number of ever described patients treated with a uniform treatment schedule and good therapeutic results, it is a significant progress in understanding of the mechanism of the disease. It might finally contribute to establishing optimal treatment standards of ocular toxoplasmosis. The different treatment we have administered lies in the aggressive initial approach followed by secondary, 6 months lasting prophylaxis introduced immediately after the intensive treatment, when a reactivation was most likely. It leads to reduction in recurrences rate and this effect is not limited to the actual time of treatment—lasts for longer.

## Introduction

A case of ocular toxoplasmosis was first described in 1923 by the Czech ophthalmologist Josef Janku [1]. The disease results from infection with the protozoan parasite *Toxoplasma gondii*, discovered by Nicole and Manceux in 1908. It was only in 1952 that the American ophthalmic pathologist Helenor Campbell Wilder-Foerster confirmed that *T*. *gondii* was a cause of retinochoroiditis. Since her pioneering publication there has been much research into ocular toxoplasmosis but in spite of considerable advances, the pathophysiology and optimal treatment of the disease have not been definitively established.

It has been estimated that 30–50% of the world population is infected with *T*. *gondii*, making it the most common parasitic infection worldwide [2, 3, 4]. Up to 80% of people with congenital toxoplasmosis will develop toxoplasmic retinochoroiditis (TRC). In acquired toxoplasmosis, TRC will develop in 1–3% of infected individuals in the USA and Europe [5, 6, 7]. In some populations and geographical locations these rates may be much higher [8, 9, 10]. When the eye is affected, toxoplasmosis follows a remitting-relapsing course. It is the most common infectious disease of the posterior eye segment as up to 50% of cases of inflammation in this region of the eye are due to *T*. *gondii* infection [7, 11]. Additionally, there are considerable differences in the epidemiology and course of TRC depending on the geographical region and the parasite type [7, 12, 13].

To date, no definitive treatment has been established, based on multicentre prospective studies of large patient populations with untreated or placebo-treated control groups as such study design would not be acceptable for ethical reasons. So far prospective studies have compared the effectiveness of different treatment regimens. There are studies which confirm the effectiveness of antiparasitic treatment in TRC but also studies which question its uses in this disease [14, 15, 16, 17].

Therefore it is of utmost importance to develop the treatment guidelines based on expert opinions [18, 19, 20, 21]. In acute focal retinochoroiditis, common systemic treatments include antifolate drugs (classically pyrimethamine and sulphonamides plus folinic acid; trimethoprim-sulfamethoxazole) or other antiprotozoal drugs (clindamycin, azithromycin, spiramycin, and atovaquone). Steroids for symptom management may be introduced with slight delay.

Reduction in the size of a postinflammatory scar following treatment with antifolates has been documented [14, 19, 20, 22, 23, 24]. Studies from Brazil reported decreased recurrence rates in patients receiving long-term treatment with low doses of antiprotozoal medications (secondary prophylaxis, SP), but the effect proved to be short-lasting and limited to the period of actual treatment [17, 18, 25, 26].

The risk for reactivation is at its highest soon after the end of a previous inflammatory event and decreases over the recurrence-free time [27, 28]. It has been documented that the risk is lower in people without pre-existing retinal scars [29, 30]. The risk for recurrence may be related to the patient age and depending on the authors it may be highest after the age of 40 years [27] or in younger individuals [29].

The purpose of this study was to evaluate the impact of the treatment regimen developed at approved at the Department of Zoonoses and Tropical Diseases (now the Department of Infectious and Tropical Diseases and Hepatology), Medical University of Warsaw on the recurrence TRC rates as well as to investigate whether other factors such as gender, age, immediate institution of intensive treatment, presence of old retinal scars and the course of inflammation including haemorrhagic lesions, retinal vascular sheathing or involvement of the optic nerve had any effect on the frequency of ocular toxoplasmosis recurrence in our material.

The adverse reactions to the treatment employed were subjected to statistical analysis, separately for the group receiving intensive treatment for 3 weeks and the group receiving secondary prophylaxis.

To our knowledge, this series of TRC patients treated according to a uniform protocol at a single institution is the largest reported to date. This is a retrospective observational study and therefore there is no control group and the results are compared to those reported from other centres.

We hope that with very good treatment outcomes achieved, our study could contribute to establishing the optimal treatment regimen and better understanding of the pathomechanisms of TRC.

## Material and Methods

The Department of Zoonoses and Tropical Diseases (now the Department of Infectious, Tropical Diseases and Hepatology), Medical University of Warsaw was at the time covered by the study one of three such referral centres in Poland, a Central European country with a population of 38 million. It admitted patients with parasitic, tropical and zoonotic diseases referred from Central Poland. At that time TRC was treated at the Department but now TRC patients receive treatment exclusively in ophthalmology departments. This retrospective study presents treatment outcomes in TRC in the years 1994–2014 measured by the recurrence rates.

The study was approved by the Bioethical Committee, Medical University of Warsaw. Anonymity of patient data was maintained.

### Patients

The patients were referred to the Department of Zoonoses and Tropical Diseases by ophthalmologists from the Clinical Departments of Ophthalmology, Medical University of Warsaw, other hospital departments of ophthalmology and community ophthalmology clinics with the presenting complaint of diminished visual acuity. The diagnosis of TRC was established by ophthalmologists according to the clinical criteria developed by Holland et al. and confirmed by serology [31]. After admission to the Department the diagnosis was confirmed by physicians experienced in the treatment of toxoplasmosis who subsequently supervised the treatment and management of the patients. When clinically required, serological tests were performed to exclude other infectious diseases such as borreliosis, cat’s scratch disease, syphilis or HIV infection. Chest X-ray and the Mantoux skin tuberculin test (PPD RT23) were used to exclude tuberculosis.

Resolution of ocular inflammation was assessed by an ophthalmologist by repeated ophthalmoscopy or slit-lamp examination evaluating the appearance of the lesion (presence of fluffy material, sharp definition of borders, amount of exudate and size of exudate granules, deposits of black pigment). Coarse granular exudate, inflammatory perivascular infiltrate, floaters and post -inflammatory membranes often persisted long after the inflammation resolved.

A recurrence was defined as a new focus of inflammation accompanied by exudate in the vitreous in a patient previously treated at the Department, with a scar in the same or fellow eye, who had already been in remission. All patients had documented changes in the retina (drawings and in some cases additionally photographs repeated regularly and covering their disease duration).

### Management/Treatment

The patients were given medication exclusively by mouth in two phases.

#### Phase 1

Intensive treatment for 21 days, instituted as soon as possible after admission to the Department. The patients received pyrimethamine 50 mg/ sulfadoxine 1000mg (P/S 50/1000) daily for the first two days, followed by pyrimethamine 25 mg/ sulfadoxine 500 mg (P/S 25/500mg) on Days 3–21, given as one tablet daily. Folinic acid was not routinely supplemented but in cases of thrombocytopenia patients were given folinic acid until their platelet counts returned to normal. Additionally, starting from Day 2 spiramycin 3 million IU three times daily was administered for 10 days followed by azithromycin 0.5 g once daily for another 6 days. From Day 2 all patients received oral prednisone, usually 40 mg in the morning. The dose was gradually tapered over 4–6 weeks depending on the resolution of the inflammation and associated exudate. Ranitidine or omeprazole were given to prevent gastro-intestinal side-effects of steroids. The patients were admitted to hospital for the first and the last 3–5 days of treatment and they were treated on an outpatient basis for at least 10 days, with a follow-up visit in the second week.

#### Phase 2

Secondary antifolate prophylaxis (A-SP) was instituted immediately after the patient completed the first phase of treatment and it did not require hospitalisation. The patients were prescribed pyrimethamine/sulfadoxine 25 mg/500 mg one tablet twice a week for 6 months without folinic acid supplementation.

When adverse reactions such as abdominal pain, hypertransaminasemia and allergies occurred in the first phase of treatment and did not resolve spontaneously, the patient could not continue the second phase of treatment. Also excluded from A-SP were patients with suspected problems with adherence to the medication regimen or potential for medication errors (e.g. elderly patients with dementia). Thrombocytopenia, usually easily reversed by omission of a few antifolate doses was not considered an exclusion criterion from A-SP.

### Laboratory Investigations/Follow-Up Visits

On first admission the following were obtained for each patient: immunoserology, full blood counts, aminotransferase levels and other blood biochemistry tests.

Until 1999 immunoserological tests for toxoplasmosis were performed by the classic indirect immunofluorescence assay (IFA) for IgG (results given in international units), and immunosorbent agglutination assay (Toxo-ISAGA bioMerieux, France) for IgM. From March 1999 until the end of the study immunoserological tests were performed by ELFA IgG, IgM (Enzyme Linked Fluorescent Assay) with VIDAS immunoanalyser (bioMerieux, France).

Full blood counts and blood biochemistry were repeated in Weeks 2 and 3 of the intensive treatment and during the 6-month A-SP full blood counts and ALT levels were obtained once monthly.

After completing the first 21-day phase of the treatment, follow-up visits were scheduled at the Outpatient Clinic at 3 months and at 6 months (at the end of A-SP), and subsequently once a year or when a recurrence was suspected. At the visit, an eye fundus examination and laboratory investigations were performed. Additionally, the patients remained under the care of their ophthalmologists locally and were assessed at least once a month for the first 6 months and then every three months or more frequently, if required.

For the purposes of this study, the review of patients’ medical records and subsequent statistical analysis covered the following three areas: (a) cases of recurrence(s); (b) no patient-reported recurrence for 10 years; (c) status at the beginning of 2014 when TRC was no longer treated at the Department. When a patient ceased to attend follow-up appointments after a few years, we assumed that there had been no reactivation since the patients had been advised to contact the Department immediately in the case of recurrence in order to undergo suitable treatment. What is important, until October 2013 no other institution in Central Poland offered treatment for TRC. Later, due to changes in the hospital profile, treatment for TRC was taken over by several hospital departments of ophthalmology and that is why any data obtained after 2013 are derived from several institutions with the resulting lack of a uniform format, smaller sizes of patient populations and difficult access to the follow-up data.

The exclusion criteria for data analysis included:

1. Premature discontinuation of any phase of treatment. The main causes were the physician’s decision to disqualify the patient from A-SP, side-effects, temporary unavailability of the study medication and the resulting need to shift to another medication and unilateral discontinuation of the treatment by the patient without their physician’s consent.
2. Immunocompromised patients treated with immunosuppressants or patients with HIV infection.
3. Patients with known autoimmune disease or with chronic viral hepatitis.
4. Patients with an uncertain diagnosis of 0T (multifocal lesions, absence of an identifiable atrophic scar after active inflammation had resolved) [27].
5. Patients with a current reactivation developed earlier than 6 weeks prior to institution of the treatment. These patients had already entered the stage of healing and therefore were not treated for acute TRC
6. Patients in whom the exact onset of current inflammation could not be identified, mostly due to an absence of macular vision (an old scar situated centrally in the macula)
7. Incomplete medical records.

**Fig 1:**
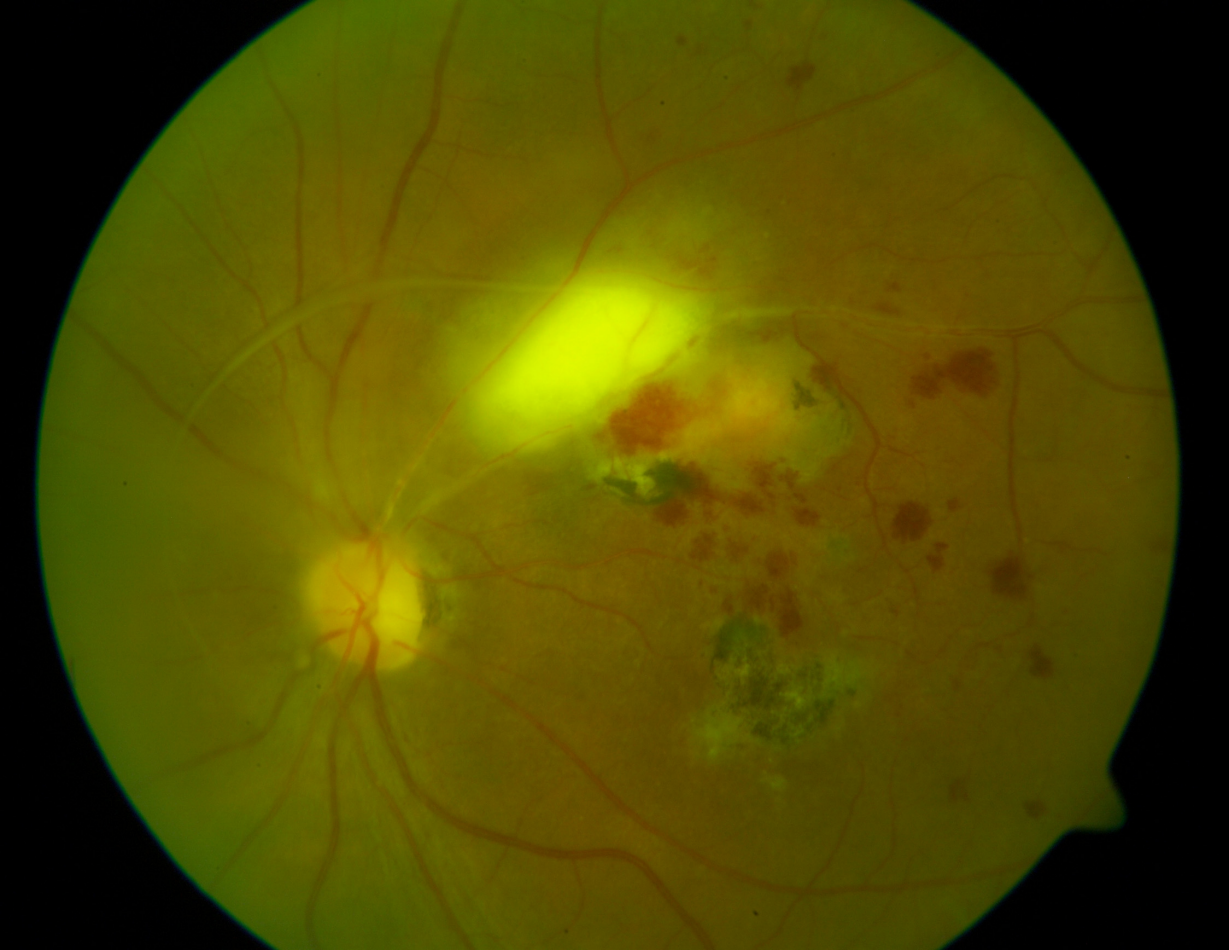
Inflammation classified as “haemorrhagic”, “with vascular sheathing”, “with a pre-existing scar” and “a focus away from the optic disc”.

**Fig 2:**
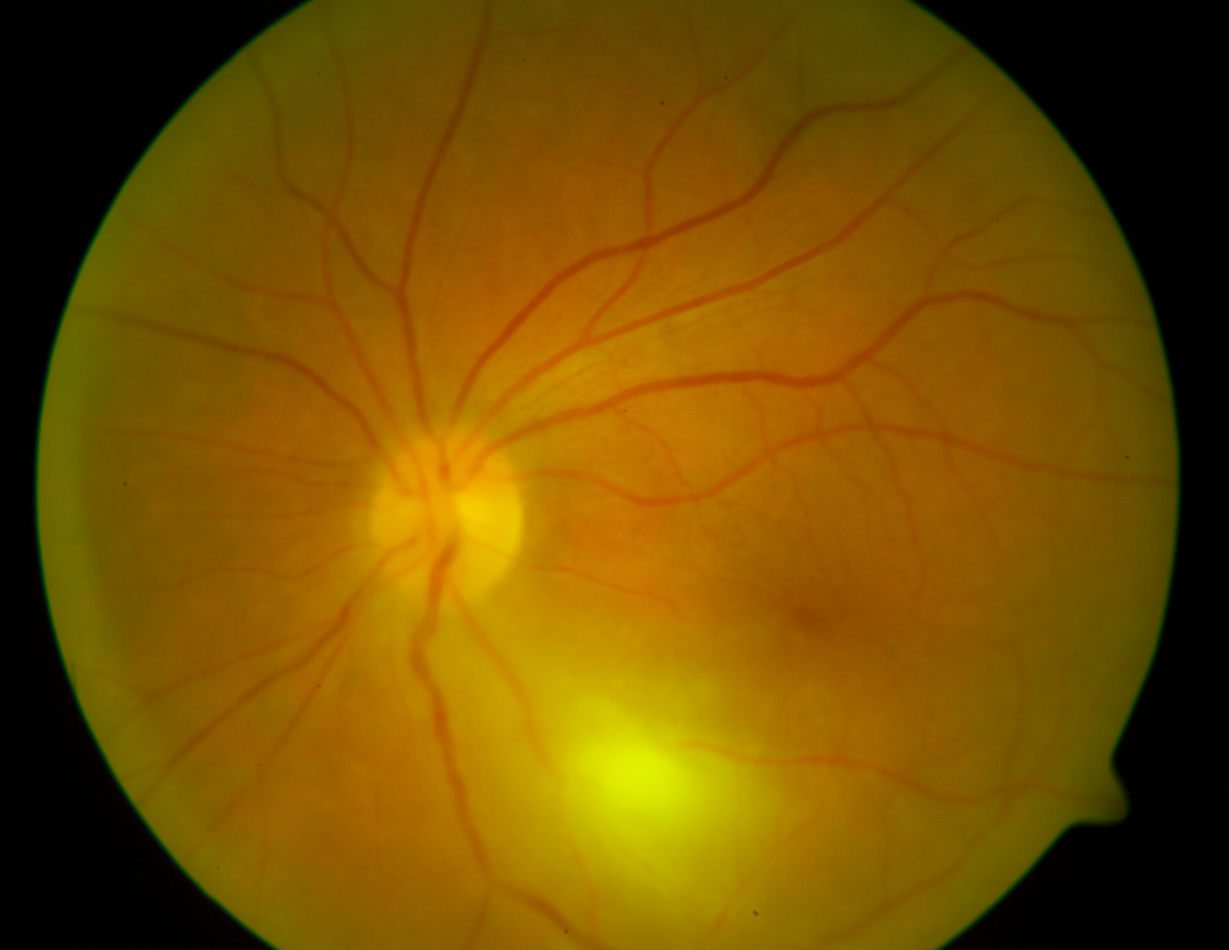
Inflammation classified as “typical”, “primary lesion” and “a focus away from the optic disc”.

The patients were subdivided into 10 groups and the impact of the following factors (each group) on the recurrence of OT was analysed:

1. Gender: females vs males.
2. Patient age: comparison between the age groups <20, 20–30, 30–40, 40–50 and > 50 years of age.
3. Presence of pre-existing scars: patients with pre-existing scars (in one or both eyes) vs patients with a first inflammation (primary lesion).
4. Patient awareness of recurrent disease (TRC): patients who gave a history of retinochoroiditis vs patients unware of their disease in the past.
5. Eye involvement: Patients with bilateral scars/foci vs patients with no scars/foci of inflammation in the fellow eye.
6. Time to treatment: number of days from first ocular manifestations to treatment: 0 to 3 days, 4 to 7 days, 8 to14 days, 15 to21 days, 21 to 42 days.
7. Haemorrhagic inflammation: patients with retinal haemorrhages vs patients without retinal haemorrhages (Fig 1).
8. Lesion location: patients with juxtrapapillary retinal lesions vs patients with lesions not directly adjacent to the optic disc.
9. Vascular sheathing/vasculitis: present vs absent (Fig 1).
10. A “typical course” of inflammation; defined as a new single fluffy focus in the previously uninvolved retina with the presence of exudate in the vitreous, with or without an old scar. The focus could be directly adjacent to the old scar but not located exclusively within the scar. The resolution of inflammation took no longer than 6–8 weeks, leaving an atrophic scar. In a “typical” inflammation there were no retinal haemorrhages or vascular sheathing and the focus was not directly adjacent to the optic nerve (see above, points 7, 8, 9). The “typical” patients were compared with the remaining patients (Fig 2).

### Statistical Methods

Descriptive statistics were given as number and percentage of patients with particular characteristics–for categorical variables and as mean and standard deviation for continuous variables.

Analysis of recurrence after first admission was done using standard survival methods. The results are presented as the Kaplan-Meier estimates of 3-year recurrence probability. The significance of potential risk factors was assessed using a log-rank test.

The analysis of time to recurrence was done using the Cox proportional hazards model with a shared gamma frailty [27, 32]. The results of univariate and multivariate analyses are presented as relative risk (RR) and adjusted relative risk (aRR) respectively.

All computations were performed using R 3.1.2 statistical software [33]. Computation of the Cox model with shared gamma frailty was done using the *frailtypack* package [34].

## Results

**Fig 3:**
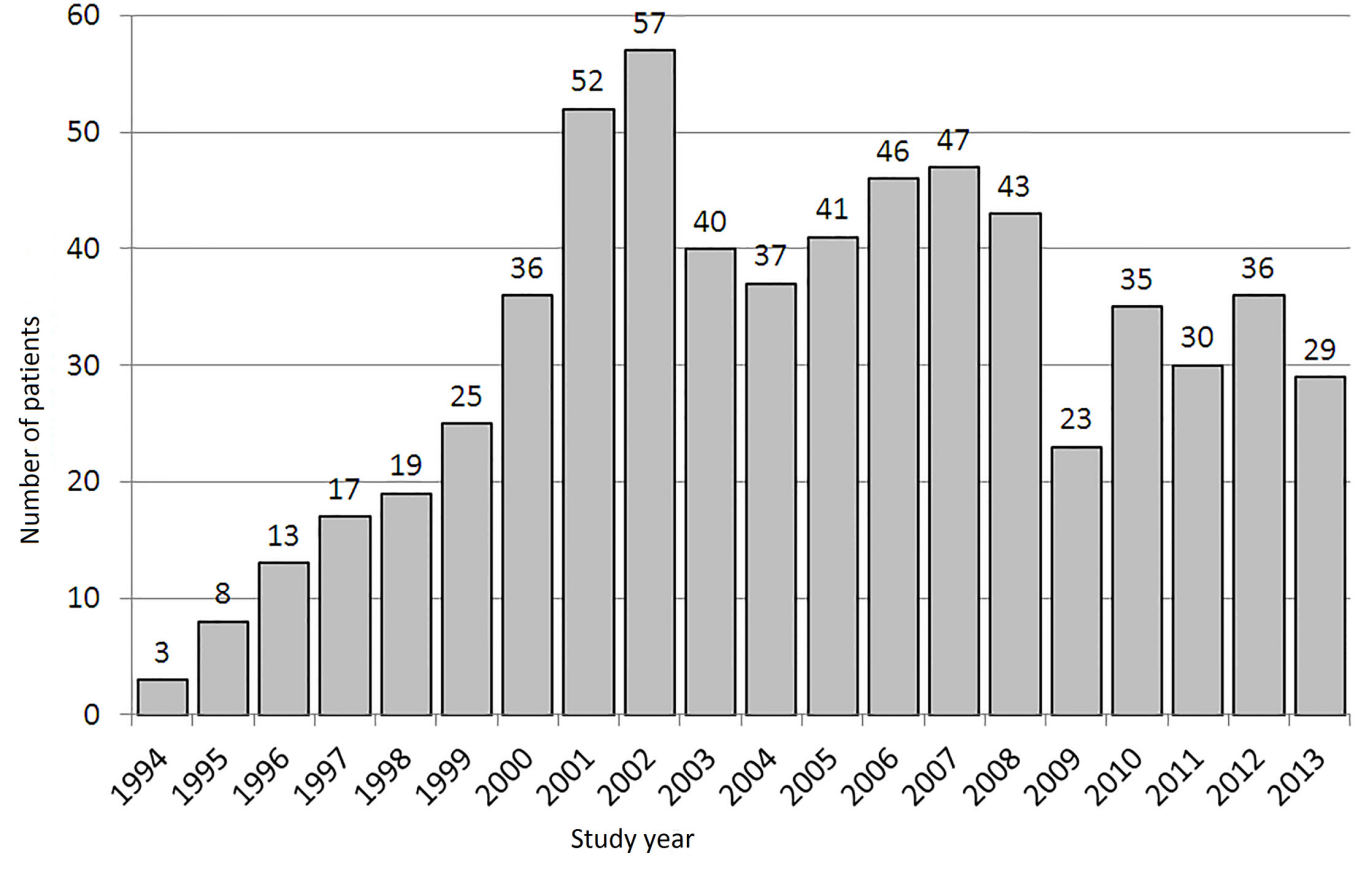
Number of patients treated in consecutive years.

The initial study population consisted of 564 patients receiving intensive treatment for 21 days at the Department of Tropical Diseases and Zoonoses from January 1994 through October 2013, followed up until the beginning of 2014. Some patients were treated more than once and the total body of data consists of 637 records, one record for each occurrence of the disease. In Fig 3 graphically we present the number of patients treated in our clinic.

There were 418 females, age range 15–83 years, mean age 35.4 (95% CI 33.9–36.9) and 219 males, age range 14–76, mean age 31.4 (95% CI 29.7–33.1). The patients’ ages ranged from 14 to 83 years, mean age: 34.0 (95% CI 32.9–35.2).

20.8% of the patients demonstrated a primary lesion, without a pre-existing scar; their mean age was 34.3 years (95% CI 30.9–37.6), whereas the mean age of those with old scars was 29.2 years (95% CI 27.8–30.5). 38.5% of the patients with pre-existing scars did not report retinochoroiditis in the past and were not aware of their disease. In most cases TRC resolved after 3–4 weeks of treatment.

Of all TRC patients only two had the serological evidence (highly positive IgM antibodies and low avidity IgG antibodies) and the clinical findings (flu-like syndrome with mild cervical lymphadenopathy) typical of acute toxoplasma invasion.

There were no cases of TCR recurrence after an interval longer than 10 years.

The treatment was administered:

(a) in 4 instances in patients in whom it was impossible to establish the actual onset of current TRC because they had no macular vision, and who were admitted to hospital after an accidental discovery of active toxoplasma ocular lesions.

(b) in 78 instances in patients in whom there was not active retinochoroiditis; they started their planned treatment from a few months to year after the last recurrence.

(c) in 124 instances in patients with manifestations of retinochoroiditis, which they developed earlier than 6 weeks prior to start of the treatment.

Groups, (a), (b) and (c) were excluded from the statistical analysis of recurrences.

(d) in 431 instances in patients with the treatment instituted within 0–6 weeks of the first ocular manifestations and those patients were included in the analysis of recurrences.

In group (d) the mean time from first ocular manifestations to treatment was 13.6 days.

Ultimately 303 patients who had received A-SP were included in the analysis of recurrences. The total body of data consisted of 352 records- one record for each occurrence of the disease.

The patient characteristics are presented in Table 1.

**Table 1:** Characteristics of patients included in the analysis of recurrences (N = 303 on first admission).

Group (classified by potential risk factor)	Values
Female gender; no, (%)	185 (61.1)
Age (years); mean + SD	30.3 ± 11.5
A pre-existing scar; no, (%)	240 (79.2)
A history of retinochoroiditis; no, (%)	148 (48.8)
Bilateral scars; no, (%)	71(23.8)
Time to treatment (days); mean +SD	13.3 ± 9.5
Haemorrhagic inflammation; n, (%)	36 (11.9)
Lesion directly adjacent to the optic disc; n, (%)	35 (11.6)
Vascular sheating /vasculitis; n, %	26 (8.6)
“Typical” inflammation; n, (%)	213 (70.3)

**Fig 4:**
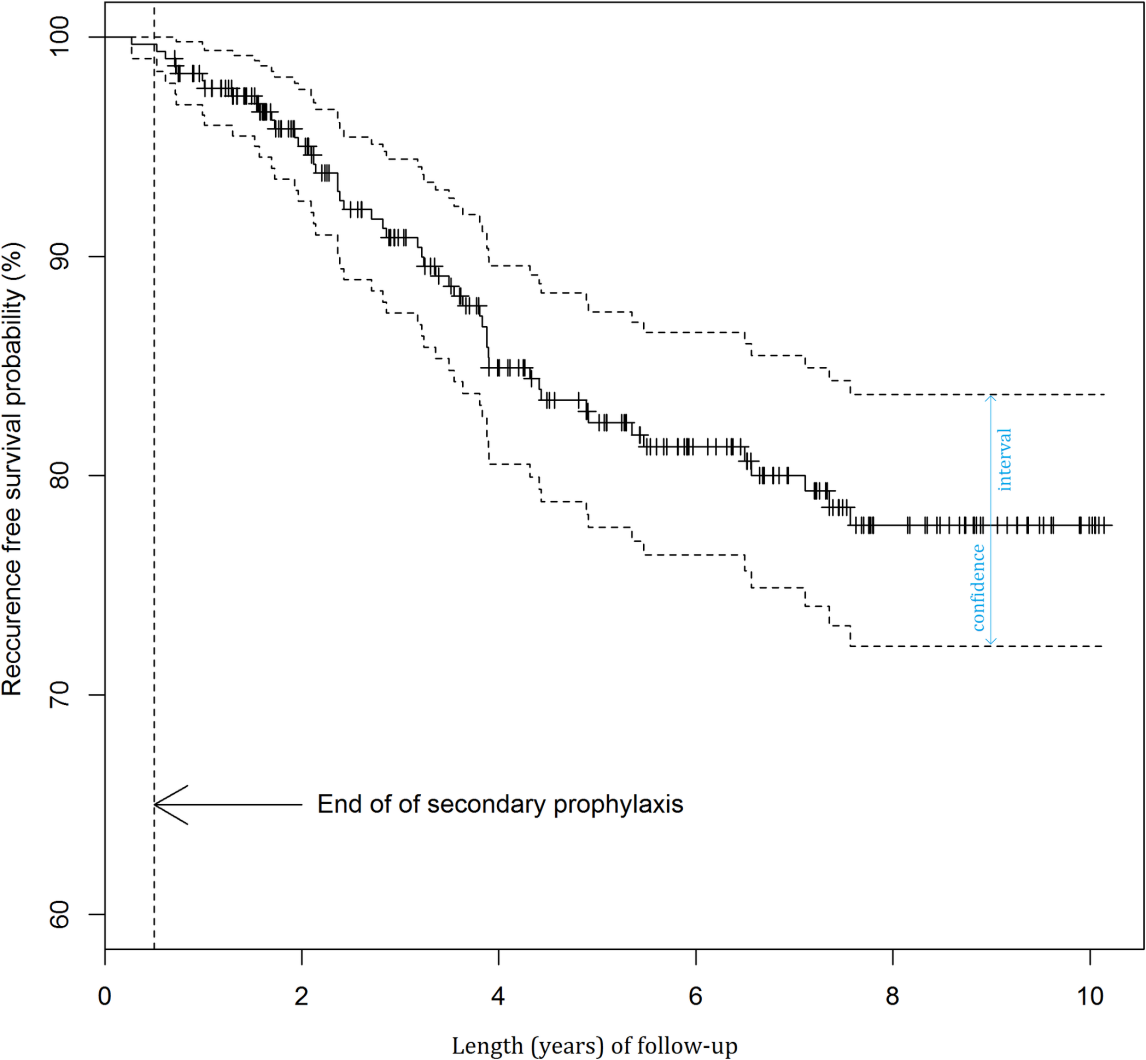
Probability of recurrence-free survival after first admission.

The results of the analysis of recurrences after first admission are presented in Fig 4 and Table 2.

**Table 2:** Kaplan-Meier estimates of 3-years probability of recurrence free survival after first admission, overall and by potential risk factors.

Group (classified by potential risk factor)	Subgroup	3-year disease-free survival n (%)	(95% CI)	P-value
Overall		303 (90.9)	(87.5–94.5)	
Gender	Females	185 (91.4)	(87.1–95.8)	0.553
	Males	118 (90.2)	(84.6–96.2)	
Age (years)	<20	59 (86.3)	(77.3–96.4)	0.259
	20–30	134 (89.7)	(84.3–95.4)	
	30–40	54 (91.6)	(84.0–99.9)	
	40–50	31 (100.0)	(100.0–100.0)	
	50–70	25 (95.7)	(87.7–100.0)	
A pre-existing scar	Present	240 (88.7)	(84.5–93.1)	0.075
	Absent	63 (100.0)	(100.0–100.0)	
A history of retinochoroiditis	Present	148 (88.7)	(83.4–94.3)	0.579
	Absent	155 (93.2)	(89.0–97.6)	
Scar location	Bilateral	71 (83.8)	(75.0–93.6)	0.701
	Unilateral	227 (92.9)	(89.3–96.6)	
Time to treatment (days)	1–3	24 (80.5)	(64.0–99.0)	0.64
	4–7	95 (93.5)	(88.2–99.2)	
	8–14	86 (89.4)	(82.3–97.2)	
	15–21	45 (95.1)	(88.6–100.0)	
	21–42	51 (88.0)	(79.4–97.5)	
Retinal haemorrhage	Present	36 (100.0)	(100.0–100.0)	0.033
	Absent	267 (89.7)	(85.8–93.7)	
Focus location	In contact with optic disc	35 (93.4)	(84.9–100.0)	0.255
	Away from optic disc	268 (90.6)	(86.9–94.4)	
Vascular sheathing/ vasculitis	Present	26 (96.2)	(89.0–100.0)	0.519
	Absent	277 (90.4)	(86.7–94.2)	
”Typical” iflammation	Present	213 (91.9)	(88.0–95.9)	0.731
	Absent	90 (88.9)	(82.3–96.0)	

**Fig 5:**
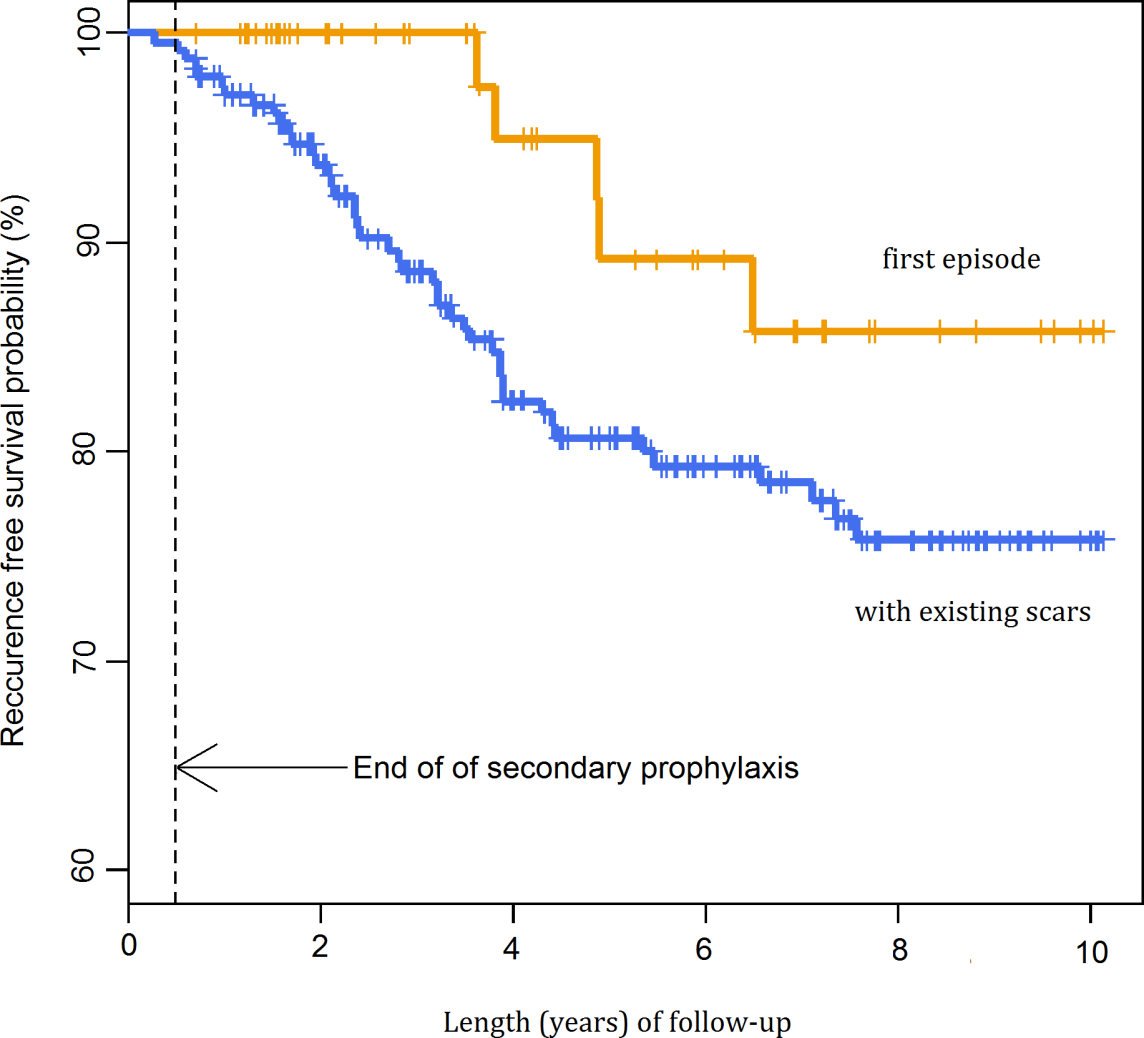
Probability of recurrence-free survival after first admission by pre-existing scars.

**Fig 6:**
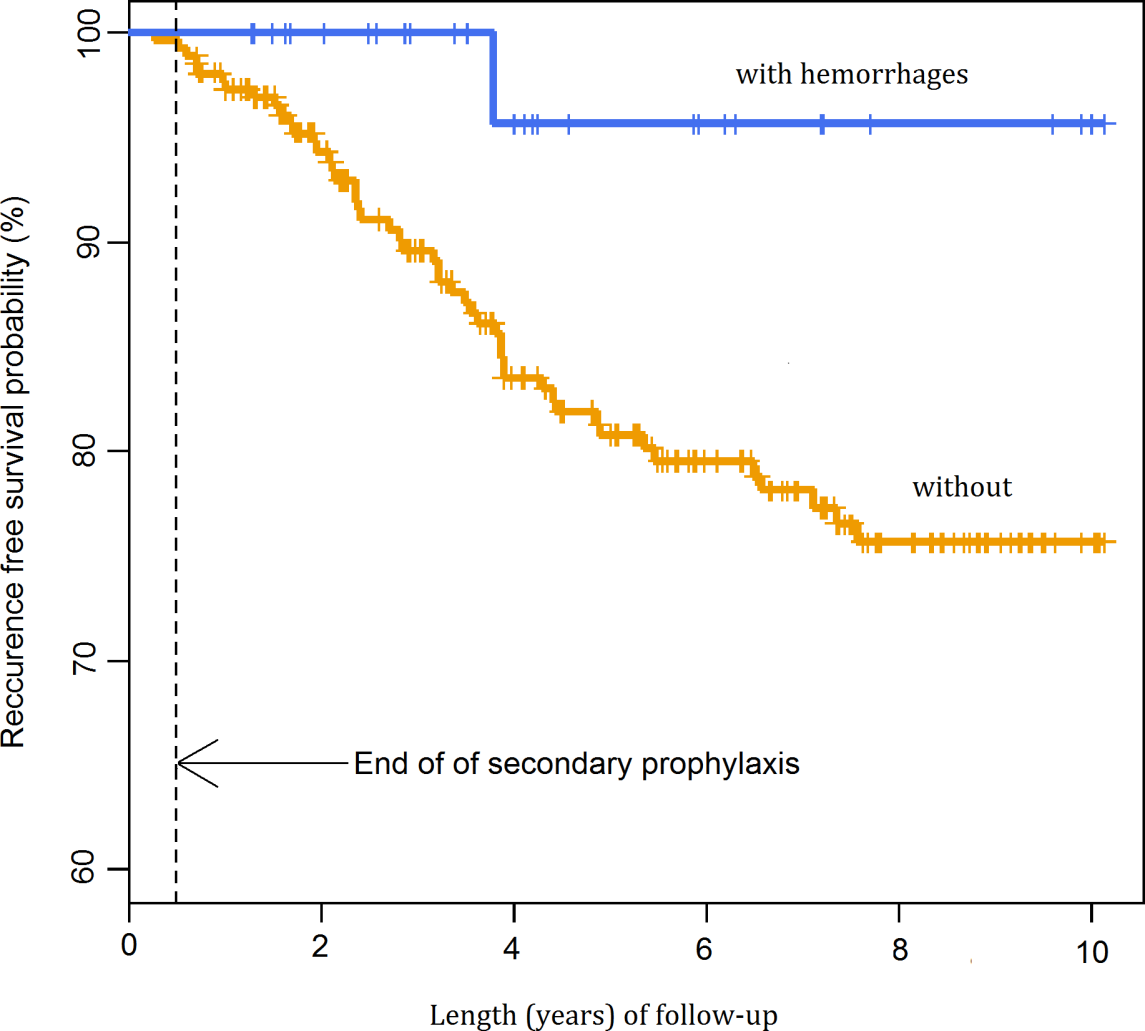
Probability of recurrence-free survival after first admission by the presence of haemorrhages.

The probability of recurrence was lower in patients without a pre-existing scar (p = 0.075) and in patients with retinal haemorrhage (p = 0.033) (Figs 5 and 6). The univariate Cox regression model demonstrated that a primary lesion (without pre-existing scars) was a statistically significant factor decreasing nearly threefold the probability of recurrence. The risk estimated in a multivariate model was 2.41-fold lower. The multivariate model also demonstrated that haemorrhagic TRC might be a factor decreasing the probability of recurrence, but the impact of this factor only approached borderline statistical significance (Table 3).

**Table 3:** Analyses of potential risk factors associated with TRC recurrence.

	Univariate model		Multivariate model	
Potential risk factor	RR (95% CI)	P-value	aRR (95% CI)	P-value
Gender: females vs males	1.03 (0.61–1.74)	0.90		
Age (1- year increment)	0.99 (0.97–1.01)	0.46		
**Pre-existing scar: present vs absent**	**2.82 (1.14–6.95)**	**0.02**	**2.41 (0.96–6.04)**	**0.06**
History of retinochoroiditis: present vs absent	1.44 (0.85–2.45)	0.18		
Pre-existing scars/foci: bilateral vs unilateral	1.14 (0.63–2.04)	0.67		
Time to treatment (1- day increment)	1.02 (0.99–1.04)	0.16		
**Retinal haemorrhages: present vs absent**	0.14 (0.01–1.64)	0.12	**0.17 (0.02–1.22)**	**0.08**
Lesion in direct contact with the optic disc: present vs absent	0.44 (0.14–1.39)	0.16		
Vascular sheathing/vasculitis: present vs absent	0.77 (0.28–2.13)	0.62		
Inflammation “typical” vs “non typical”	0.97 (0.56–1.66)	0.91		

**Fig 7:**
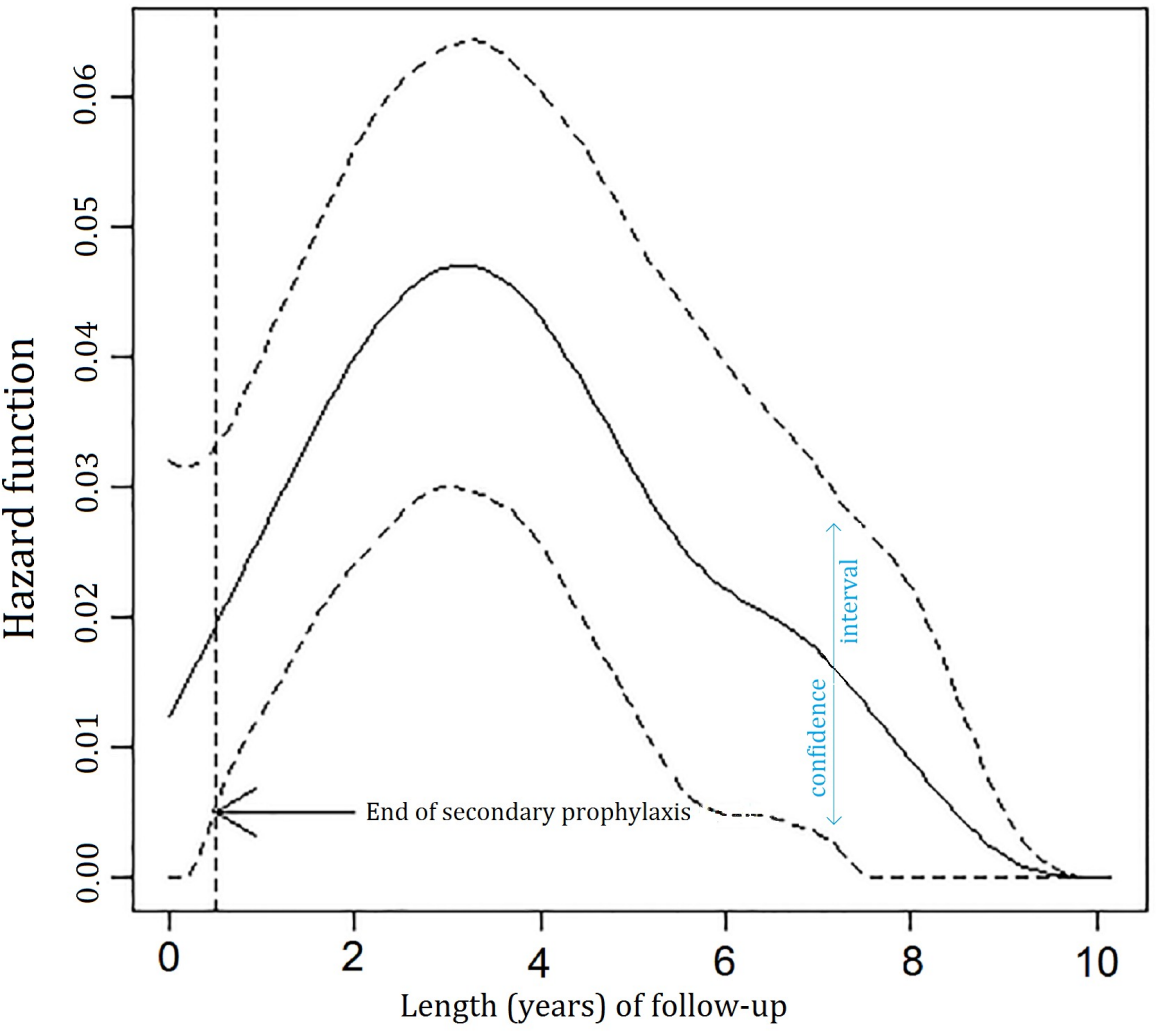
Hazard function (95% CI) derived from the final multivariate model.

The multivariate Cox regression model demonstrated that the risk for recurrence was low in the short period after disease onset, reached the maximum after approximately 3.5 years and subsequently decreased over time (Fig 7).

**Fig 8:**
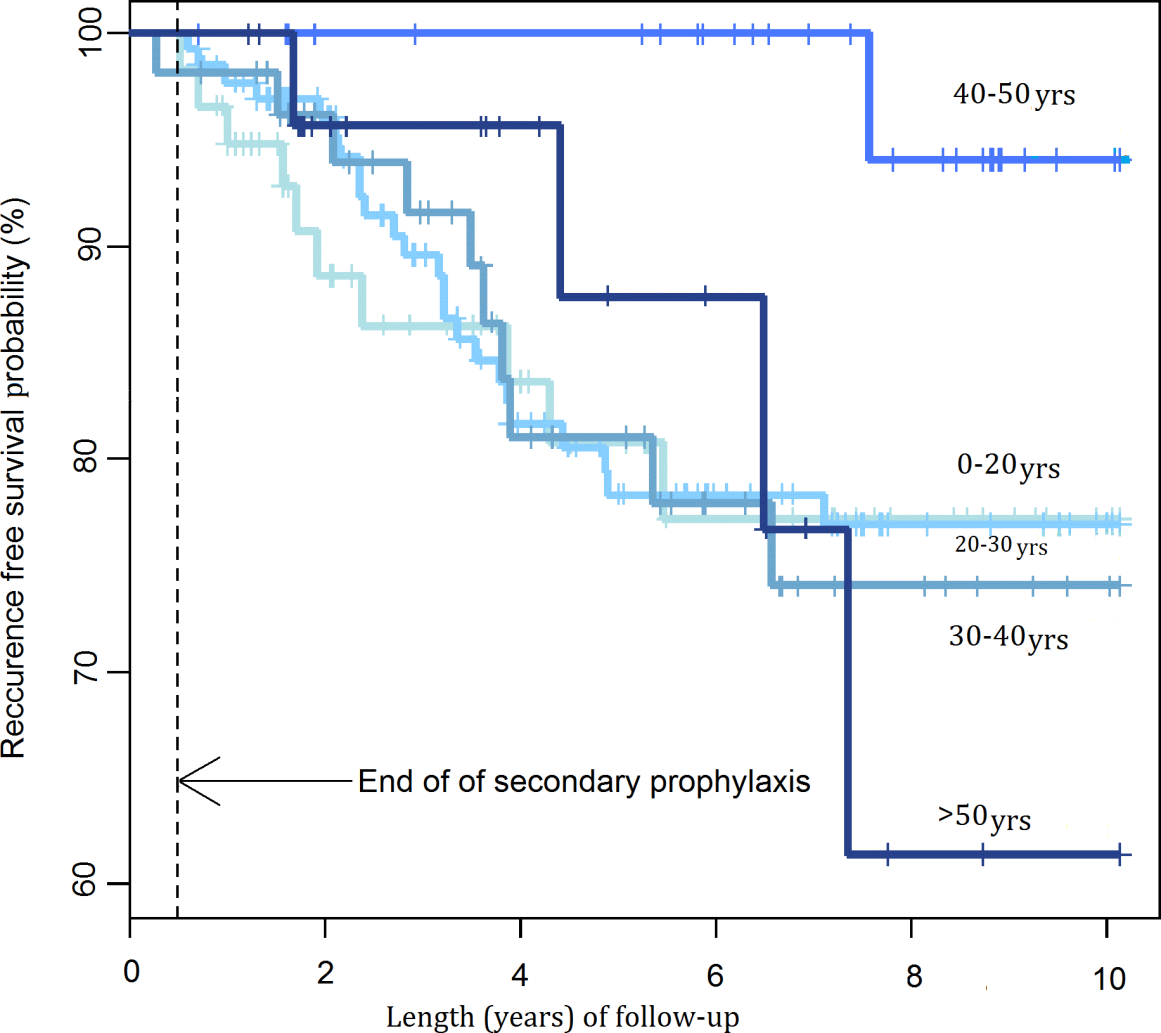
Probability of recurrence-free survival after first admission by age groups.

Gender, patient age, a past history of retinochoroiditis, bilateral ocular lesions, time to treatment, location of the focus of inflammation directly adjacent to the optic nerve, vascular sheathing and a “typical course” of inflammation had no statistically significant impact on the recurrence rates (Fig 8).

The mostly commonly reported adverse reactions in the first intensive phase of the treatment were hypertransaminasemia (24,6% of patients), thrombocytopenia (8,3%), morbiliform drug skin eruption (3%), abdominal pain (1,4%) and Stevens-Johnson syndrome (0,3%). 2.2% of patients discontinued treatment due to adverse reaction.

During A-SP adverse reactions were observed in 2,3% of patients who subsequently discontinued treatment.

## Discussion

### Background

For a patient with recurrent TRC the most important treatment outcomes are visual acuity and the frequency of recurrences. In our study we did not assess visual acuity, duration of reactivation or size of the resulting scar since these parameters largely depend on the location of the focus of inflammation, previous damage to the macula and maculopapular bundle or the size of a pre-existing scar. For the purposes of this study we accepted the frequency of recurrences as an objective and simple measure of treatment effectiveness and all calculations are related to this particular outcome.

### How the Treatment Regimen Was Developed

The Department of Tropical Diseases and Zoonoses had earlier experience in both prophylaxis and treatment of malaria with pyrimethamine and sulfadoxine (P/S) and we decided to use this therapeutic combination in the treatment for toxoplasmosis, including TRC. In most cases an intensive antifolate treatment of TRC for three weeks was sufficient for the retinal inflammation to resolve. Myelosuppression, with decreased platelet counts as its earliest manifestation, was extremely rare during treatment and routine folinic acid supplementation was not considered necessary in all patients. To increase the therapeutic effectiveness without increasing the dosage and length of P/S treatment, antiprotozoal agents spiramycin and azithromycin were administered in combination with P/S [35]. Spiramycin poorly penetrates across biological membranes and that is why after administration for 10 days it was replaced with azithromycin. Azithromycin easily penetrates across the blood-ocular barrier even when a “sealed” ultrastructure has been formed. Cases of relapses were observed in Week 4, i.e. soon after completion of 3-week intensive antifolate treatment. In such cases, antifolates had to be reintroduced, but at lower doses similar to those used in the prophylaxis of malaria. In 1994 a uniform treatment regimen was introduced for TRC patients which included 6-month secondary prophylaxis with antifolates twice weekly and it was successfully used for the next 20 years.

Initially, in the 1990s, such treatment regimen was used in nearly all TRC patients referred by ophthalmologists. Later it was decided not to include those patients who presented late when the disease had almost resolved clinically. There was no clinical justification for the intensive treatment and exposing the patients to its potential adverse effects.

### Results

When compared to the treatment outcomes reported by other authors using A-SP (Silveira et al. [17], Felix et al.[18]), we observed a similar length of recurrence-free period during A-SP. In our material the decreased probability of recurrence was not limited to the A-SP period as described by Silveira et al. in 2015, but it was observed for longer periods of time [25].

The analysis of recurrence free-survival after first admission showed the probability of a 3-year recurrence-free interval in 90.9% of patients, a significantly higher percentage than reported by Tungal-Tutkun et al. [36] and Reich et al. [30] or Garweg et al. (if the interval between the primary lesion and the first reactivation is considered) [29, 22]. None of the authors, however, used A-SP and some patients did not receive any antiparasitic drugs [37].

The curve of recurrence risk differs from that given by Holland et al. [27]. With the institution of A-SP immediately after the intensive treatment for TRC, i.e. when a reactivation was most likely, there was no recurrence during A-SP. Following A-SP the recurrence rates were low and recurrence-free periods tended to be longer. In our series one patient only experienced a recurrence during A-SP, which demonstrates that secondary prophylaxis offers protection in the period when recurrences are most likely. Immediately after the end of A-SP the probability of recurrence was very low and it began to slowly increase, being delayed and reaching its maximum at 3.5 years (Fig 7). After that time, the risk for recurrence steadily decreased over time similar to the study by Holland et al. [27] None of the Dutch patients described by Holland et al. received A-SP and a third of them did not take any antiparasitic drugs [37].

The effect of A-SP at the time of its administration most likely consists of inhibiting the multiplication of tachyzoites released from the rupturing tissue cysts. The mechanism of a decreased recurrence incidence 3.5 years after A-SP is more difficult to explain. Obviously the number of tissue cysts must be reduced, but antifolates act on tachyzoites, not on tissue cysts, the only exception being atovaquone active against tissue cysts *in vitro*. If SP with antifolates had actually reduced the number of tissue cysts, then the effect of SP described in the study of Silveira et al. would have continued beyond the period covered by SP. It may be hypothesized that asymptomatic formation of new cysts lasts longer than clinical inflammation. At that stage, SP acts on the dynamic equilibrium that is somewhat disturbed and the cyst turnover is increased [38, 39].

In this study, pre-existing retinal scars significantly increased the probability of recurrence in agreement with the findings of Garweg et al. and Reich et al. while Holland et al. did not observe this association [27, 29, 30]. It could be expected that patients with a history of (frequent) recurrences would be likely to develop reactivation in the future statistically more frequently than patients with less frequent or no previous recurrences. We wanted to investigate this assumption by comparing a group of patients who gave a history of recurrence(s) vs patients without a previous recurrence and found no confirming evidence. Interestingly, nearly 40% of the patients with a pre-existing scar, compared to two-thirds of patients in the study of Tungal–Tutkun et al. [36] were not aware of their disease.

In patients with haemorrhagic retinochoroiditis (small haermorrhages from damaged blood vessels) there were statistically fewer recurrences after such incidents (a weak correlation). This might be due to the intensity of inflammation which boosted a specific immune response in the patients.

It seemed that vascular sheathing and vasculitis similar to the foci of inflammation located directly adjacent to the optic nerve would be related to more frequent recurrences, but actually no such effect was observed.

We did not find any differences in the frequency of recurrences between genders, but female patients predominated in our series. The same was observed by Garweg et al. and Reich et al., but not by Tungal-Tutkun et al. [29, 30, 36]. One possible explanation is that women pay more attention to their health and tend to attend scheduled follow-up visits more regularly.

The effect of patient age on the frequency of recurrences has been demonstrated by a number of studies, but the findings are not consistent as some authors report a higher probability of recurrence in younger individuals and some claim that reactivation is more likely in older patients. We did not find any correlation between age and recurrence frequency but we studied the association between age and recurrence in the whole series, without subdivision into groups with a first incident and with a subsequent incident (Fig 8). In patients with a first incident of TRC, recurrences were less frequent in patients aged 30 years and older, but due to a small size of the group it could be subdivided into two age groups only (ages <30 years and > 30 years), the sizes being too small for meaningful statistical analysis (p = 0.411).

We observed some beneficial, though not statistically significant, effect of an earlier introduction of treatment on the frequency of recurrences. To our knowledge, to date none of the studies of TRC recurrences has taken into consideration the impact of delayed treatment. In our opinion, intensive treatment should be instituted as soon as possible, optimally within the first 3 days of the first ocular manifestation. A delay in treatment may have a significant effect on treatment outcomes such as visual acuity, scar size or complications, but probably a negligible impact on the frequency of recurrences.

In our material, the mean time to treatment was approximately 13 days after first ocular manifestation (when we excluded those who started their treatment longer than 6 weeks after first manifestations), when the inflammation was at its peak and had already caused irreversible retinal damage. We suspect that treatment was probably began as late at other centres and that is why in many it could not produce significant improvement of visual acuity. We were able to institute the treatment within the first 1–3 days of ocular manifestations in a limited number of patients only. In those cases the inflammation resolved rapidly, the resulting scars were very small and superficial and there were no massive exudates in the vitreous body [22].

In our material two patients only had the clinical and serological features of a recent invasion. The number was similar to the study of Tungal-Tutkum et al. but much smaller than in most other studies, most likely due to the exclusion from the present analysis of patients who did not form atrophic scars after TRC [36, 37]. Such type of inflammation in the course of TRC was described by other authors at the stage of acute primary invasion [40].

Adverse reactions to treatment were subjected to statistical analysis and the result will be presented in detail in a separate paper (manuscript in preparation). According to the preliminary assessment in the phase of intensive treatment hypertransaminasemia was the most common adverse reaction and Stevens-Johnson syndrome—the most dangerous and the least common. Most of the adverse reactions were not severe and very seldom required discontinuation of the treatment. In most cases transaminases were only mildly elevated and hypertransaminasemia was always reversible. We did not identify the possible causative drug(s) in the combination used, i.e. antibiotics, anifolate drugs and steroids. Thrombocytopenia with antifolate treatment seems to be the major safety concern in many studies, but in this patients population it was rarely reported, even when folinic acid supplementation was not used. Abdominal pain always was transient, and most likely due to steroids. Hypersensitivity reactions, including Stevens-Johnson syndrome which is rarely reported in this context were probably caused by sulfonamides. Generally, although the overall incidence of adverse reactions to intensive treatment was high, the reactions were mild and transient and with careful monitoring the treatment was safe.

During A-SP adverse events were rare, but invariably required discontinuation of the treatment.

### Limitations of the Study

The main limitation of the study which we have identified is the assumption that if a patient did not report to the Department in 10 years, they did not experience any recurrences in that period. That might not have been true, but was imposed by the observational character of the study. In fact, it was not verified whether the patients were alive or had not emigrated to mention but a few explanations. Considering that all patients were advised to report to the Department in the case of any ocular problems and the population was relatively young, the impact of these limitations on the findings seems negligible. In the period covered by the study we were the only institution offering treatment for TRC in the region and patients were routinely referred by ophthalmology departments. Another limitation may be due to recognizing any cases of focal retinitis without a residual scar as of doubtful etiology and their exclusion from the analysis.

The present study, although there was no control group, confirms the effectiveness of the proposed treatment of ocular toxoplasmosis and secondary antifolate prophylaxis instituted soon after the active inflammation. The ideal treatment regimen, drugs and their dosage should be determined by further studies. Patient compliance (adherence) when they have to take one P/S (or similar antifolates) tablet twice weekly for 6 months is good, their everyday functioning is not affected and the quality of life may be actually improved.

## References

[1] Janku J. [Patogenesa a patologicka anatomie tak nazvaného vrozeného kolobomu zluté skvmy v oku normalne velikem a mikrophtalmickém s nalazem parazitu v sitnici]. Cas Lék Ces 1923;62:1021–7.

[2] Flegr J, Prandota J, Sovickova M, Israili ZH. Toxoplasmosis- a global threat. Correlation of latent toxoplasmosis with specific disease burden in a set of 88 countries. PLoS One 2014; 9(3):e90203 10.1371/journal.pone.0090203 24662942 PMC3963851

[3] Robert-Gangneux F, Darde ML. Epidemiology of and diagnostic strategies for toxoplasmosis. Clin Microbiol Rev 2012;25(2):264–96. 10.1128/CMR.05013-11 22491772 PMC3346298

[4] European Food Safety Authority. The European Union Summary Report on Trends and Sources of Zoonoses, Zoonotic Agents and Food-borne Outbreaks in 2012. EFSA Journal 2014;12(2):3547:209–17. 10.2903/j.efsa.2018.5500 PMC7009540 32625785

[5] Wallon M, Kodjikian L, Binquet C et al Long-term ocular prognosis in 327 children with congenital toxoplasmosis. Pediatrics 2004;113 (6):1567–72. 15173475 10.1542/peds.113.6.1567

[6] Gilbert RE, Stanford MR. Is ocular toxoplasmosis caused by prenatal or postnatal infection? Br J Ophthalmol 2000;84(2):224–6. 10655202 10.1136/bjo.84.2.224 PMC1723371

[7] Holland GN. Ocular toxoplasmosis: a global reassessment. Part I: epidemiology and course of disease. Am J Ophtalmol 2003;136(6):973–88. 10.1016/j.ajo.2003.09.040 14644206

[8] Bowie WR, King AS, Werker DH et al Outbreak of toxoplasmosis associated with municipal drinking water. Lancet 1997;350(9072):173–7. 9250185 10.1016/s0140-6736(96)11105-3

[9] Vaudaux JD, Muccioli C, James ER et al Identification of an atypical strain of *Toxoplasma gondii* as the cause of waterborne outbreak of toxoplasmosis in Santa Isabel do Ivai, Brazil. J Infect Dis 2010;202(8):1226–33. 10.1086/656397 20836703 PMC5718918

[10] Holland GN. An epidemic of toxoplasmosis: lessons from Coimbatore, India. Arch Ophthalmol 2010;128(1):126–8. 10.1001/archophthalmol.2008.538 20065229

[11] Jones JL, Holland GN. Annual burden of ocular toxoplasmosis in the US. Am J Trop Hyg 2010;82(3):464–5. 10.4269/ajtmh.2010.09-0664 PMC2829910 20207874

[12] Commodaro AG, Belfort RN, Rizzo LV et al Ocular toxoplasmosis: an update and review of the literature. Mem Inst Oswaldo Cruz 2009;104(2):345–50. 19430662 10.1590/s0074-02762009000200030

[13] Pleyer U, Schulter D, Manz M. Ocular toxoplasmosis: recent aspects of pathophysiology and clinical implications. Ophtalmic Res 2014;52:116–23. 10.1159/000363141 25248050

[14] Bosch-Driessen LH, Verbraak FD, Suttorp-Schulten MS et al A prospective, randomized trial of pyrimethamine and azithromycin vs pyrimethamine and sulfadiazine for the treatment of ocular toxoplasmosis. Am J Ophthalmol 2002;134(1):34–40. 12095805 10.1016/s0002-9394(02)01537-4

[15] Gilbert RE, See SE, Jones LV, Stanford MS. Antibiotics versus control for toxoplasma retinochoroiditis. Cochrane Database Syst Rev 2002;(1):CD002218 11869630 10.1002/14651858.CD002218

[16] Stanford MR, Gilbert RE. Treating ocular toxoplasmosis: current evidence. Mem Inst Oswaldo Cruz 2009;104(2):312–5. 19430659 10.1590/s0074-02762009000200027

[17] Silveira C, Belfort R Jr, Muccioli C et al The effect of long-term intermittent trimethoprim/sulfamethoxazole treatment on recurrences of toxoplasmic retinochoroiditis. Am J Ophthalmol 2002;134(1)41–6. 12095806 10.1016/s0002-9394(02)01527-1

[18] Felix JP, Lira RP, Zacchia RS, Toribio JM, Nascimento MA, Arieta CE. Trimethoprim-sulfamethoxazole versus placebo to reduce the risk of recurrences of *Toxoplasma gondii* retinochoroiditis: randomized controlled clinical trial. Am J Ophthalmol 2014;157(4):762–6. 10.1016/j.ajo.2013.12.022 24388839

[19] Wakefield D, Cunningham ET Jr, Pavesio C, Garweg JG, Zierhut M. Controversies in ocular toxoplasmosis. Ocul Immunol Inflamm. 2011;19(1):2–9. 10.3109/09273948.2011.547157 21250922

[20] Kim SJ, Scott IU, Brown GC et al Interventions for toxoplasma retinochoroiditis: a report by the American Academy of Ophthalmology. Ophthalmology 2013;120(2):371–8. 10.1016/j.ophtha.2012.07.061 23062648

[21] Holland GN, Lewis KG. An update on current practices in the management of ocular toxoplasmosis. Am J Ophthalmol 2002;134(1):102–14. 12095816 10.1016/s0002-9394(02)01526-x

[22] Rothova A, Meenken C, Buitenhuis HJ et al Therapy for ocular toxoplasmosis. Am J Ophthalmol 1993;115(4):517–23. 8470726 10.1016/s0002-9394(14)74456-3

[23] Soheilian M, Sadoughi MM, Ghaiarnia M et al Prospective randomized trial of trimethoprim/sulfamethoxazole versus pyrimethamine and sulfadiazine in the treatment of ocular toxoplasmosis. Ophthalmology 2005;112(11):1876–82. 16171866 10.1016/j.ophtha.2005.05.025

[24] Garweg JG, Stanford MR. Therapy for ocular toxoplasmosis- the future. Ocul Immunol Inflamm.2013;21(4):300–5. 10.3109/09273948.2013.779724 23617277

[25] Silveira C, Muccioli C, Nusselblatt R, Belfort R Jr. The Effect of Long-term Intermittent Trimethoprim/Sulfamethoxazole Treatment on Recurrences of Toxoplasmic Retinochoroiditis:10 Years of Follow-up. Ocul Immunol Inflamm 2015;23(3):246–7. 10.3109/09273948.2014.964422 25325434

[26] Harrell M, Carvounis PE. Current treatment of toxoplasma retinochoroiditis: an evidence-based review. J Ophthalmol 2014;2014:273506 10.1155/2014/273506 25197557 PMC4147351

[27] Holland GH, Crespi CM, ten Dam-van Loon N et al Analysis of recurrence patterns associated with toxoplasmic retinochoroiditis. Am J Ophthalmol 2008;145(6):1007–13. 10.1016/j.ajo.2008.01.023 18343351

[28] de-la-Torre A, Rios-Cadavid AC, Cardozo-Garcia CM, Gomez-Marin JE. Frequency and factors associated with recurrences of ocular toxoplasmosis in a referral centre in Colombia. Br J Ophthalmol 2009;93(8):1001–4. 10.1136/bjo.2008.155861 19429576

[29] Garweg JG, Scherrer JN, Halberstadt M. Recurrence characteristics in European patients with ocular toxoplasmosis. Br J Ophthalmol 2008;92(9):1253–6. 10.1136/bjo.2007.123661 18211930

[30] Reich M, Ruppenstein M, Becker MD, Mackensen F. Time patterns of recurrences and factors predisposing for a higher risk of recurrence of ocular toxoplasmosis. Retina 2015;35(4):809–19. 10.1097/IAE.0000000000000361 25299969

[31] Holland GN, O’Connor GR, Belfort R Jr, Remington JS. Toxoplasmosis In: Pepose JS, Holland GN, Wilhelmus KR, (1996) Ocular Infection and Immunity. St Louis, Missouri: Mosby-Year Book, Inc,:1183–1223

[32] Rondeau V, Mathoulin-Pelissier S, Jacqmin-Gadda H, Brouste V, Soubeyran P. Joint frailty models for recurring events and death using maximum penalized likelihood estimation: application on cancer events. Biostatistics 2007;8(4):708–21. 17267392 10.1093/biostatistics/kxl043

[33] The R core team. R: A language and environment for statistical computing Vienna, Austria: R Foundation for statistical computing, 2009.

[34] Rondeau V, Mazroui Y, Gonzalez JR. frailtypack: An R Package for the Analysis of Correlated Survival Data with Frailty Models Using Penalized Likelihood Estimation or Parametric Estimation. J StatSoftw 2012;47:1–28.

[35] Derouin F, Almadany F, Chau F, Rouveix B, Pocidalo JJ. Synergistic activity of azithromycin and pyrimethamine or sulfadiazine in acute experimental toxoplasmosis. Antimicrob Agents Chemother 1992;36(5):997–1001. 1324642 10.1128/aac.36.5.997 PMC188824

[36] Tugal-Tutkun I, Corum I, Otuk B, Urgancioglu M. Active ocular toxoplasmosis in Turkish patients: a report on 109 cases. Int Ophthalmol 2005;26(6):221–8. 17318320 10.1007/s10792-007-9047-8

[37] Bosch-Driessen LE, Berendschot TT, Ongkosuwito JV, Rothova A. Ocular toxoplasmosis: clinical features and prognosis of 154 patients. Ophthalmology 2002;109(5):869–78. 11986090 10.1016/s0161-6420(02)00990-9

[38] McHugh TD, Bathgate T, Mangan J, Johnson JD, Holliman RE, Butcher PD. Recognition of tissue cyst-specific antigens in reactivating toxoplasmosis. J Med Microbiol 1997;46(7):587–95. 9236743 10.1099/00222615-46-7-587

[39] Gormley PD, Pavesio CE, Minnasian D, Lightman S. Effects of drug therapy on toxoplasma cysts in an animal model of acute and chronic disease. Invest Ophthalmol Vis Sci 1998;39(7):1171–5. 9620076

[40] Holland GN, Muccioli C, Silveira C, Weisz JM, Belfort R Jr, O’Connor GR. Intraocular inflammatory reactions without focal necrotizing retinochoroiditis in patients with acquired systemic toxoplasmosis. Am J Ophthalmol 1999;128(4):413–20. 10577581 10.1016/s0002-9394(99)00300-1

